# The Informational Dynamics of Mean‒Variance Relationships in Fertilizer Markets: An Entropic Investigation

**DOI:** 10.3390/e20090677

**Published:** 2018-09-06

**Authors:** Salim Lahmiri, Stelios Bekiros

**Affiliations:** 1 Department of Quantitative Methods, ESCA School of Management, Casablanca 20000, Morocco; 2 Department of Accounting & Finance, Athens University of Economics & Business, 76 Patission Str., GR10434 Athens, Greece; 3 Department of Economics, European University Institute (EUI), 50121 Florence, Italy

**Keywords:** fertilizer market, informational dynamics, risk‒return trade-off, EGARCH-M, global financial crisis, entropy, C40, G15, H12

## Abstract

The risk‒return trade-off is a fundamental relationship that has received a large amount of attention in financial and economic analysis. Indeed, it has important implications for understanding linear dynamics in price returns and active quantitative portfolio optimization. The main contributions of this work include, firstly, examining such a relationship in five major fertilizer markets through different time periods: a period of low variability in returns and a period of high variability such as that during which the recent global financial crisis occurred. Secondly, we explore how entropy in those markets varies during the investigated time periods. This requires us to assess their inherent informational dynamics. The empirical results show that higher volatility is associated with a larger return in diammonium phosphate, potassium chloride, triple super phosphate, and urea market, but not rock phosphate. In addition, the magnitude of this relationship is low during a period of high variability. It is concluded that key statistical patterns of return and the relationship between return and volatility are affected during high variability periods. Our findings indicate that entropy in return and volatility series of each fertilizer market increase significantly during time periods of high variability.

## 1. Introduction

There are abundant studies on various commodity markets in terms of causal linkages, efficiency, and predictability. For instance, Fernandez [1] examined linear and non-linear Granger causality in U.S. price indices and commodity series, Fernandez [2] examined the influence of nominal returns and real price cycles of various commodities, Giuliodori and Rodriguez [3] analysed the relation between European, China and U.S. prices of stainless steel market, Ahmed et al. [4] conducted an empirical investigation to determine the long- and short-term relationships between natural resource abundance and economic growth in the Iranian economy, and Jain and Biswal [5] explored the relationships between global prices of gold, crude oil, the USD‒INR exchange rate, and the stock market in India. More recently, Kanjilal and Ghosh [6] investigated the dynamic relationship of global crude oil and gold prices, while Lahmiri et al. [7] proposed a general framework for measuring short- and long-term dynamics in asset classes between financial and commodity markets and also examined efficiency in these markets [8].

Moreover, significant attention has been given to studying volatility and volatility transmission in commodity markets. For example, Batten et al. [9] investigated the macroeconomic determinants (business cycle, monetary environment and financial market sentiment) in price volatilities of four precious metals (gold, silver, platinum and palladium prices). Limited evidence was found that the same macroeconomic factors jointly influence the volatility processes of the four precious metal price series. However, there is evidence of a volatility feedback between the precious metals. Ma [10] examined the impact of the change in forward pricing mechanism on the volatility of iron ore spot prices. Empirical results indicated evidence of a long memory and an asymmetrical effect for the volatility of the iron ore spot price, and that the change in the forward pricing may reduce the volatility of the spot price. In addition, only negative shocks have an effect on the volatility of the iron ore spot price. Todorova et al. [11] modelled volatility spillovers between five of the most liquid and important non-ferrous metals contracts (aluminium, copper, lead, nickel, and zinc). The authors illustrated the presence of volatility transmission in metal markets and concluded that most pronounced volatility spillovers are identified in the longer term. Gil-Alana [12] analysed volatility persistence and leverage effect across six non-ferrous metals spot and futures series in India and showed evidence of volatility persistence and leverage effects. Tiwari and Sahadudheen [13] examined the impact of oil price volatility on gold price volatility and found that positive and negative shocks have a different magnitude of effect on gold prices. Singhal and Ghosh (2016) [14] investigated the time-varying co-movements between crude oil and Indian stock market returns, both at the aggregate and the sector level. The empirical results showed that during crises time-varying correlations increased significantly, but post-2011 they reached the pre-crisis level. Yaya et al. [15] analysed volatility persistence in the prices of oil and gold after the global financial crisis. It was revealed that the volatility persistence in the gold market is lower than that of the oil market and that there is an oil‒gold volatility spillover. More recently, Lahmiri and Bekiros [16] examined the efficiency in volatility of various financial and commodity markets. Their empirical findings demonstrated that complexity increased during the crisis period, but diminished during the pre-crisis period. Also, the global financial crisis affected market complexity and nonlinearity particularly concerning volatility.

It is worth mentioning that, in recent years, there has been a growing interest in studying fertilizer markets. For example, Geman and Eleuterio [17] studied the validity of investing capital in fertilizer-mining companies from a market return perspective for individual and institutional investors. It was shown that corn, wheat and fertilizer prices generated extremely high returns during January 2004–December 2007. Lahmiri [18] studied cointegration and causal linkages among five different fertilizer markets before and during the recent global financial crisis. Fertilizer markets are closely linked to each other during low and high regimes. Particularly during high regimes (after crisis), causality effects have emerged and impulse responses are higher after a crisis. Finally, Lahmiri [19] investigated asymmetry, leverage, and the persistence of shocks on the price volatility of fertilizers before and after the 2007 international financial crisis. It was concluded that, after the international financial crisis, the statistical characteristics of each type of fertilizer price changed, volatilities increased, and responses to shocks were more pronounced.

The purpose of the current work is to examine the effect of price volatility on price return in major world fertilizer markets. Obviously, such an investigation has considerable economic implications for both importers and exporters of fertilizers, as well as for fertilizer traders. Our work aims to contribute to the existing literature on fertilizer markets in six different ways. First, the relationship between volatility and returns in such a key market of the global agriculture industry is investigated. Second, our empirical investigation considers all five major markets of fertilizers, namely rock phosphate (RP), triple super phosphate (TSP), diammonium phosphate (DAP), urea, and potassium chloride (PC). Third, the relationship between volatility and returns in each fertilizer market is investigated during periods of low and high volatility. Fourth, we realized that the risk‒return relationship in fertilizer markets may arise from economic shocks or speculative activities. Fifth, our study will contribute to the limited works on fertilizer markets and enrich the literature on the volatility of commodity markets. Finally, we investigate how Shannon entropy [20] in return and volatility series of the global fertilizer markets vary across low and high variability time periods. Indeed, such an investigation would reveal how the market agent information content and flow within those markets is affected at different regimes. This is an important step towards understanding their inherent informational dynamics. The entropic measures we used were successfully employed in other financial applications [21,22,23,24,25,26,27]. Shannon entropy is employed in this work due to its simplicity and popularity in financial applications [21,22,23,24,25,26,27]. 

The reminder of this paper is as follows: Section 2 presents our methodology, and Section 3 provides the empirical results. Finally, Section 4 discusses our work and concludes.

## 2. Methodology

In our work, to investigate the return‒volatility relationship in fertilizer markets, the exponential generalized autoregressive conditional heteroscedasticity (EGARCH) model [28] is employed to estimate volatility in return series. Indeed, the advantage of the EGARCH model is its ability to account for the time-varying volatility process with asymmetric responses to both positive and negative price changes. In addition, contrary to the standard GARCH process, the EGARCH model does not impose positive constraints on the estimated parameters to ensure the positivity of the estimated conditional variance series. Furthermore, employing the EGARCH model allows for avoiding possible misspecifications in the volatility process [29]. Then, to gauge the relationship between return and volatility series, the ARMA-EGARCH-in-mean (ARMA-EGARCH-M) model is estimated. For instance, let *r_t_* denote the return series and *r_t_* = log(*p_t_*) − log(*p_t_*_−1_), where *p* is the fertilizer commodity price and *t* is the time script. Then, let us assume that the return series *r_t_* of a given fertilizer commodity follows an autoregressive moving average (ARMA) model with AR of order *p* and MA of order *q*. Afterward, the ARMA-EGARCH-M can be expressed in mathematical form as:

(1) $$r_{t}=\mu +\sum_{i=1}^{p}\phi_{i}r_{t-1}+\sum_{j=1}^{q}\theta_{i}\varepsilon_{t-1}+\lambda h_{t}+\varepsilon_{t}$$

(2) $$\log\left(h_{t}^{2}\right)=\omega +\sum_{g=1}^{m}\beta_{g}\log\left(h_{t-g}^{2}\right)+\sum_{l=1}^{n}\alpha_{l}\left|\frac{\varepsilon_{t-l}}{h_{t-l}}\right|+\sum_{k=1}^{o}\gamma_{k}\frac{\varepsilon_{t-k}}{h_{t-k}}\;$$

(3) $$\varepsilon ∼S.t\left(0,h_{t},v\right),$$

where *h* is the conditional variance; *μ*, *φ*, *θ*, and *λ* are the mean equation (Equation (1)) parameters to be estimated. Also, *ω*, *β*, *α*, and *γ* are the variance equation (Equation (2)) parameters to be estimated. Finally, *ε* is the error term assumed to follow a Student’s-*t* (*S*.*t*) distribution with zero mean, conditional variance *h*, and *v* degrees of freedom. Indeed, we supposed that the error term obeys a Student-*t* (*S*.*t*) distribution to accommodate any potential presence of skewness and excess kurtosis in each fertilizer return series. The ARMA-GARCH-M model includes a heteroskedasticity term (*h_t_*) in the mean equation (Equation (1)) to show the impact of volatility on return series. In other words, in the mean equation (Equation (1)), the parameter *λ* captures the relationship between conditional volatility *h_t_* and return *r_t_*. Recall that the log transformation in the left-hand of the variance equation (Equation (2)) allows a remarkable flexibility for EGARCH estimation as it ensures that the conditional variance *h_t_* is positive. In this regard, non-negativity restrictions on parameters in the variance equation are not explicitly imposed. In the EGARCH framework, the parameter *γ* captures asymmetry in volatility. In particular, conditional volatility *h_t_* is asymmetric when *γ* ≠ 0; and, negative shocks cause more volatility than positive ones when *γ* < 0. All parameters are estimated by using the quasi-maximum likelihood (QML) technique according to the following log-likelihood function:

(4) $$\log L\left(\varepsilon \right)=-0.5\sum_{t=1}^{T}\left[\log\left(2\pi \right)+\log\left(h_{t}\right)+\varepsilon_{t}^{2}/h_{t}\right],$$

where *T* is the sample size. In our paper, the orders *p* and *q* of ARMA model in Equation (1) and orders *m*, *n*, and *o* of EGARCH model in Equation (2) are all restricted between one and three. Then, optimal values were determined based on the lowest Akaike Information Criterion (AIC).

Finally, Shannon entropy [20] is employed to quantify randomness, stochasticity and informational flows in both return and volatility series of each market. If we consider the following signal *X* = ${\left\{x_{i}\right\}}_{i=1}^{n}$, then Shannon entropy (*SE*) is expressed as:

(5) $$SE\left(X\right)=-\sum_{i=1}^{n}p\left(x_{i}\right)\;\log p\left(x_{i}\right),$$

where *p*(*x_i_*) = Prob(*X* = *x_i_*) is a discrete probability such that $\sum_{i}p_{i}=1$. Shannon entropy reaches its maximum when all values of the underlying informational signal ${\left\{x_{i}\right\}}_{i=1}^{n}$ are equally probable. In particular, when it comes close to log(*n*), ${\left\{x_{i}\right\}}_{i=1}^{n}$ is nearly random. Conversely, Shannon entropy approaches a minimum score when a particular *x_i_* is guaranteed to happen, for instance with *p*(*x_i_*) = 1. In this work, *p*(*x_i_*) is computed as follows:

(6) $$\hat{p}\left(x_{i}\right)=\frac{L_{i}}{n},$$

where *L_i_* denotes how often the value *x_i_* appears in the available signal and *n* is the length of the signal or number of samples in the signal. Recall that the probability given in Equation (6) is a naive one, but it is the simplest approximation of the probability associated with the value *x_i_*. Thus, *SE*(*X*) in Equation (5) can be rewritten as:

(7) $$SE\left(X\right)=SE_{naive}(X)=-\sum_{i=1}^{n}\frac{L_{i}}{n}\;\log\left(\frac{L_{i}}{n}\right).$$

In this work, a histogram with equal-width bins is adopted to estimate the probabilities in Equation (6). The number of bins is set to 10 as this properly covers the range of return values and shows the shape of the underlying distributions. Useful details on Shannon entropy estimation and applications can also be found in [25,26,27].

## 3. Data and Results

**Figure 1:**
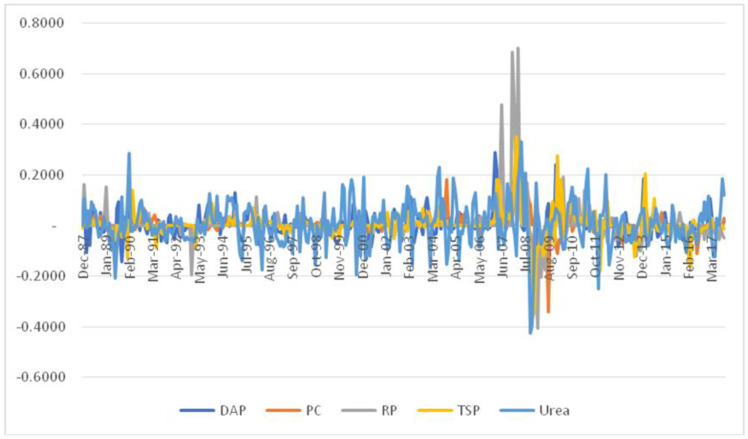
Evolution of log-returns in each fertilizer market.

**Figure A1:**
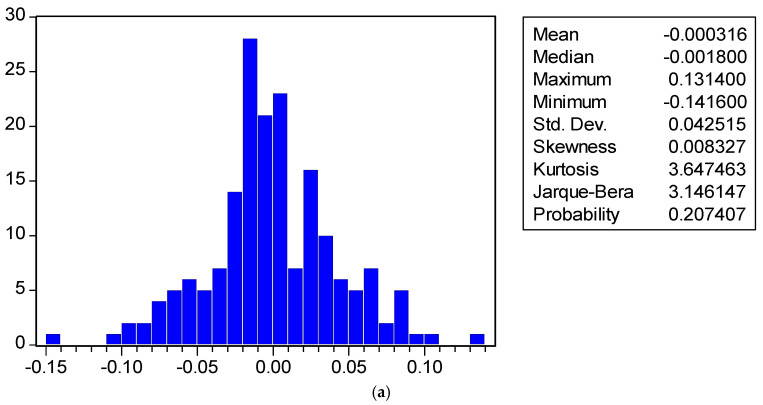

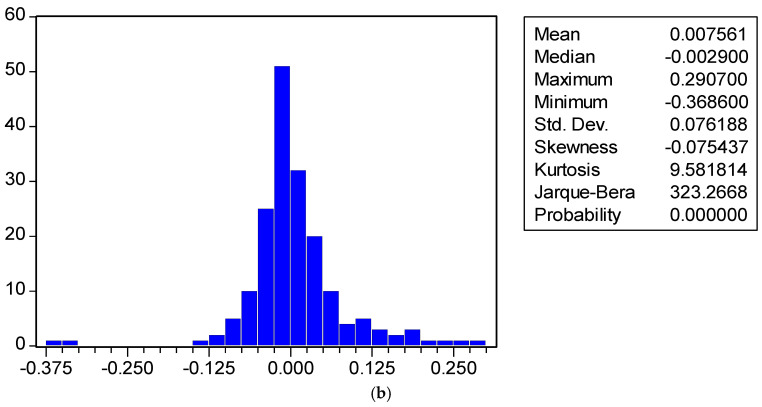
(**a**) Histogram and descriptive statistics of DAP market during a low variability period; (**b**) Histogram and descriptive statistics of DAP market during a high variability period.

**Figure A2:**
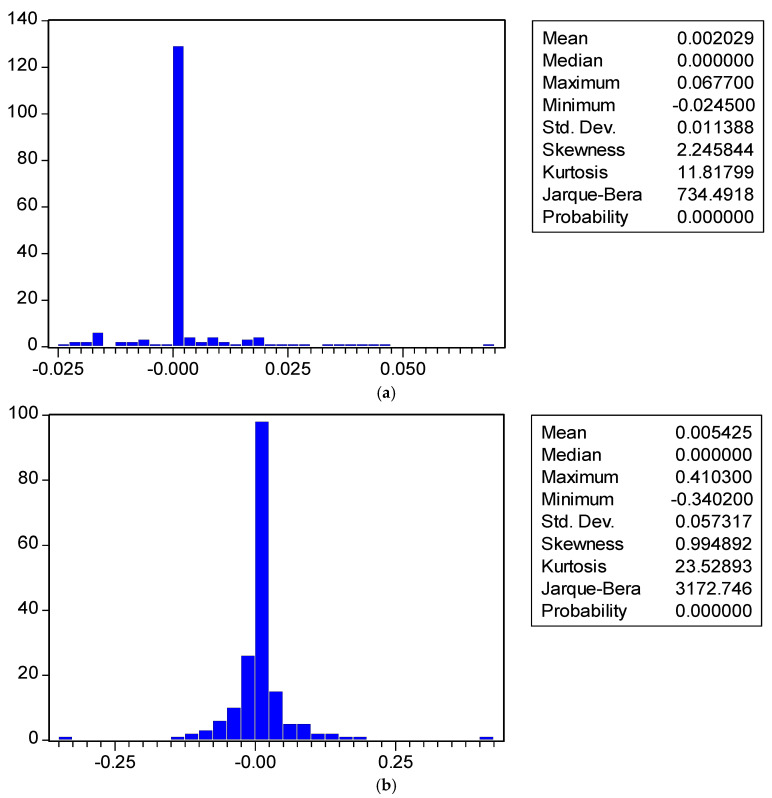
(**a**) Histogram and descriptive statistics of PC market during a low variability period; (**b**) Histogram and descriptive statistics of PC market during a high variability period.

**Figure A3:**
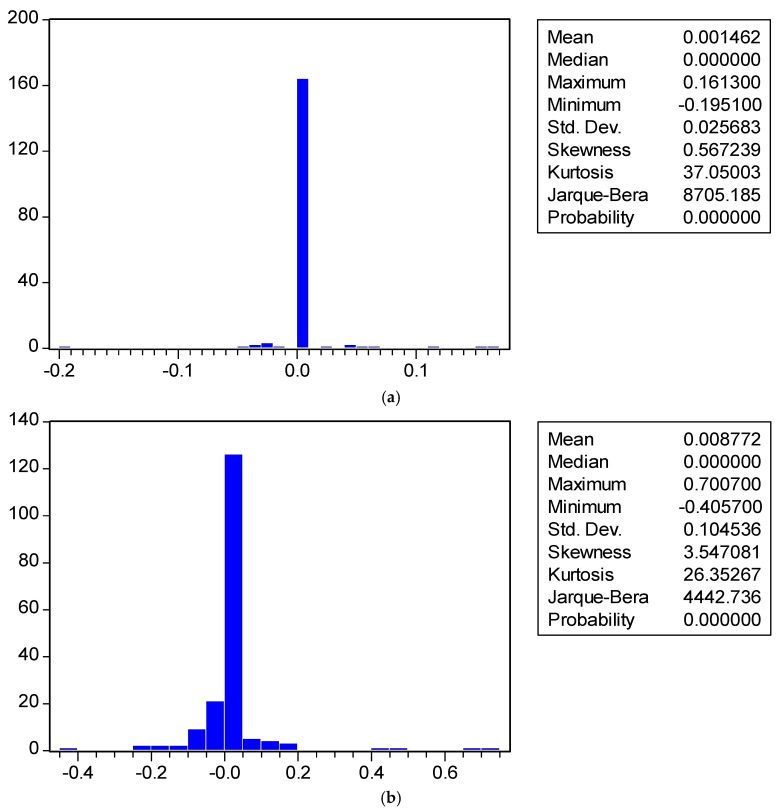
(**a**) Histogram and descriptive statistics of RP market during a low variability period; (**b**) Histogram and descriptive statistics of RP market during a high variability period.

**Figure A4:**
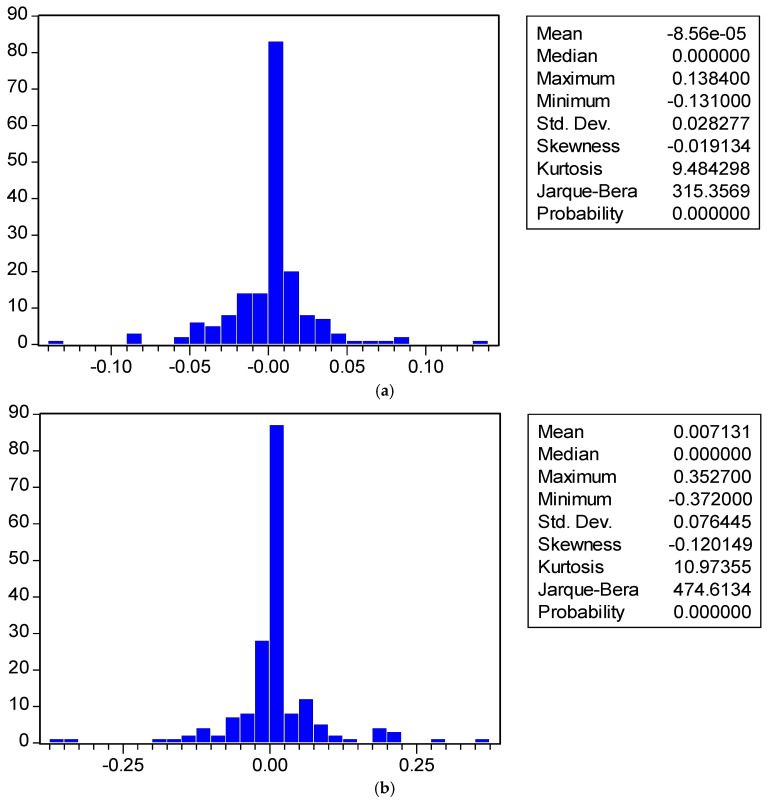
(**a**) Histogram and descriptive statistics of TSP market during a low variability period; (**b**) Histogram and descriptive statistics of TSP market during a high variability period.

**Figure A5:**
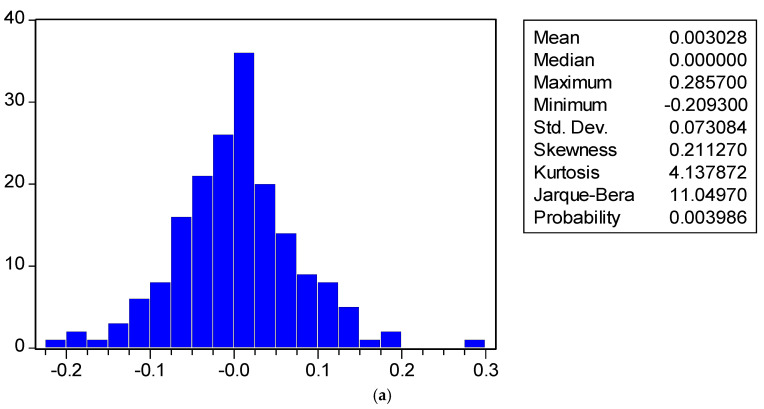

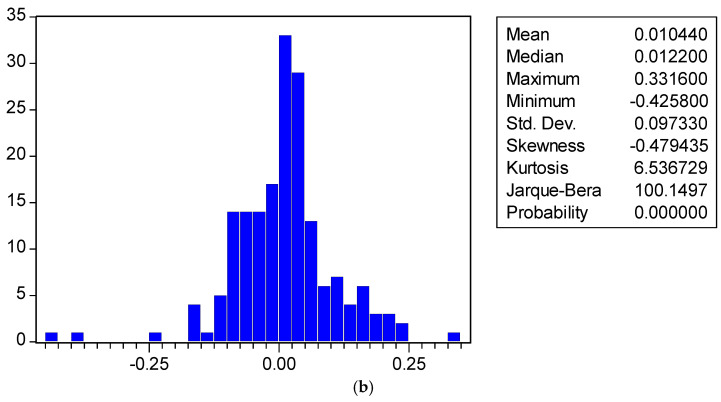
(**a**) Histogram and descriptive statistics of urea market during a low variability period; (**b**) Histogram and descriptive statistics of urea market during a high variability period.

We gathered available monthly data from IndexMundi on rock phosphate (RP), triple super phosphate (TSP), diammonium phosphate (DAP), urea, and potassium chloride (PC). The full sample spans from December 1985 to October 2017, i.e., 359 observations. It is divided into two subsamples: December 1985 to October 2003, covering 179 observations, and November 2003 to December 2017, with 180 observations. The first subsample corresponds to low variability in return of all five commodities. On the contrary, the second subsample corresponds to remarkable higher variability in all return series. Particularly, low and high variability time periods are characterized by a noticeable increase in standard deviation of return series in all fertilizer markets. Recall that the second subsample includes the most recent global financial crisis. For illustration purposes, the evolution of price return of each market is depicted in Figure 1. Moreover, the histogram and main descriptive statistics during low variability and high variability periods for DAP, PC, RP, TSP, and urea are displayed in Figure A1, Figure A2, Figure A3, Figure A4 and Figure A5, respectively, in the Appendix A. Specifically, it is interesting to observe that the kurtosis statistic significantly increased during high variability times, except for the RP market. Additionally, the skewness statistic markedly decreased during high variability sample periods in all fertilizer markets. Also, the distributions represented by histograms are different across low and high variability time periods.

The empirical results on the relationship between return and volatility in fertilizer markets are reported in Table 1. Specifically, for each fertilizer market and for each period, the estimated value of the coefficient *λ* from the mean equation (Equation (1)) (which is used to measure the effect of volatility on return) and its associated probability-value (*p*-value) are both computed and provided. Additionally, Table 1 provides values and *p*-values of all other estimated parameters of Equations (1) and (2).

As shown in Table 1, the estimated parameter (coefficient) *λ* is positive in all markets and time periods, reflecting a positive risk‒return relationship, except in the rock phosphate market during both the first and second subsamples and in the triple super phosphate market during the second subsample. In addition, the estimated parameter *λ* is statistically significant at the 5% level only in the case of the DAP market during the first subsample (a low variability time period). Furthermore, *λ* takes values ranging from −5.6948 (for the rock phosphate market during a low variability time period) to 4.6759 (for the potassium chloride market through a low variability time period). Moreover, by looking at absolute values of the estimated parameter *λ*, one can see that the size is lower during the second time period than the first. In general, the obtained results indicate that the risk‒return relationship in fertilizer markets is positive and not statistically significant. In addition, the relationship is weak during high variability time periods in comparison with low variability time periods.

**Figure 2:**
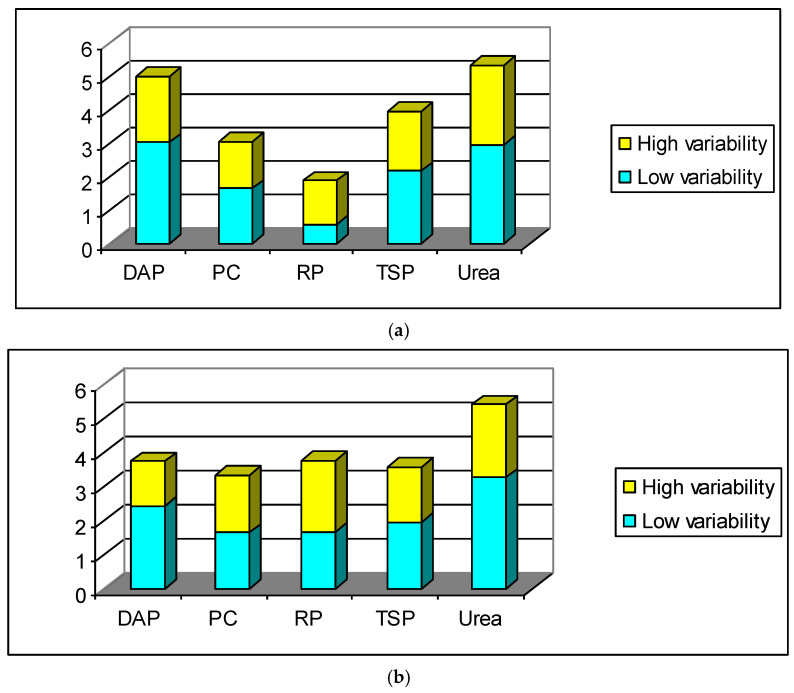
(**a**) Shannon entropy measurement for return series; (**b**) Shannon entropy measurement for volatility series.

Furthermore, Figure 2a,b exhibit the measured entropy of return and volatility series for each fertilizer market. For both categories of signals, the main significant fact is that entropy is remarkably higher during the high variability period than in the low variability one. Indeed, by visual inspection of Figure 2a,b we observe that the value of entropy (given in yellow) during high variability time periods is much higher than its corresponding value (given in blue) during low variability sample periods. In this regard, high volatility conditions are characterized by an increase in uncertainty about future agent expectations, which significantly affects the informational content when making forecasting or investing decisions.

## 4. Discussion and Conclusions

The relationship between risk and return is one of the major topics in quantitative finance, where such an association is investigated in various major international stock markets [30,31,32,33]. In order to enrich the existing literature on international finance, and more specifically on fertilizer markets [17,18,19], in this article we investigated the relationship between volatility and return in five major fertilizer markets during two different time periods: a period of low variability in returns and a period of high variability coinciding with the most recent global financial crisis. In this vein, this is the first article to examine the effect of volatility on return in such commodity markets, to the best of our knowledge. In this regard, the flexible EGARCH-M model was employed under the assumption that the error term obeys a Student’s-*t* distribution due to the presence of skewness and excess kurtosis in each fertilizer return series.

In theory, return increases with volatility; hence, the sign of the parameter *λ* used to capture the risk‒return relationship is expected to be positive. This concept is verified during all time periods for all markets, except for the phosphate rock market. Indeed, the empirical findings showed that the risk‒return relationship in fertilizer markets is positive in all markets and through both low and high variability time periods, except in the rock phosphate market during both low and high variability time periods and in the triple super phosphate market throughout the high variability period. In general, the results can be summarized as follows: the effect of risk on return in fertilizer markets is positive, not statistically significant, and weak during period of high variability in price.

Our empirical findings clearly show that, except for phosphate rock, higher volatility is associated with larger return in diammonium phosphate, potassium chloride, triple super phosphate, and urea. The magnitude of this relationship is low during high variability in these markets. This low impact of volatility on returns could be explained by the recent global financial crisis. In general, our empirical finding that volatility positively affects return in four fertilizer markets is in accordance with a standard financial theory [34] stating that the risk‒return relationship is positive; in other words, risk has rewards. In addition, it was found that the returns distribution in each market was profoundly changed through high variability periods. Our results are in line with previous works where it was found that the recent global financial crisis profoundly affected cointegration and causal linkages among fertilizer markets [18], and also affected how asymmetry, leverage, and the persistence of shocks impact on the price volatility of fertilizers [19].

Lastly, our entropy-based results indicated that a high variability time period reveals more randomness in price expectations and deduced volatility, which means that less information is available to agents during market stress. This salient finding suggests that high variability is characterized by a hike in stochasticity and disorder in the information content, especially of asset returns, which in turn negatively affects the level of volatility. On the contrary, the relative stability in the financial markets of fertilizers signifies the availability of ample information and hence low variability in return predictability. Specifically, we observed that entropy in urea returns and volatility is the highest among all markets during both low and high variability time periods, followed by the DAP market. This implies fewer information flows in the urea (and DAP) market compared to the others. Interestingly, our entropic investigation was able to capture the degree of inherent uncertainty and randomness in fertilizer markets and also to highlight the differences between them.

The main policy implication emanating from this investigation is that the magnitude of dynamics of return reaction to volatility in fertilizer markets changes with the level of market uncertainty. Therefore, policy makers, importers and exporters should all consider the changing effect of volatility on return in fertilizer markets during times of financial crisis.

## Figures and Tables

**Table 1:** Estimation results.

	First Subsample		Second Subsample		Full Sample	
	Value	*p*-Value	Value	*p*-Value	Value	*p*-Value
DAP						
*μ*	−0.0192	0.0181	−0.0093	0.1551	−0.0154	0.0053
*φ*	0.0677	0.6333	0.1744	0.1231	0.1299	0.1767
*θ*	0.4465	0.0005	0.5317	0.0000	0.4878	0.0000
*λ*	0.5535	0.0358	0.1255	0.4908	0.3810	0.0268
*ω*	−0.8732	0.0308	−2.8214	0.0004	−2.3303	0.0000
*β*	0.3266	0.0166	1.2059	0.0062	0.7685	0.0000
*α*	−0.1515	0.0945	−0.2859	0.2613	−0.2278	0.0403
*γ*	0.9065	0.0000	0.6302	0.0000	0.7108	0.0000
PC						
*μ*	−0.0548	0.1687	−0.0000	0.9306	−0.0001	0.4559
*φ*	−0.3773	0.0269	0.3126	0.0100	0.0967	0.3474
*θ*	0.3276	0.2095	0.0482	0.7314	0.1556	0.1486
*λ*	4.6759	0.7115	0.0006	0.6758	0.0059	0.2427
*ω*	−8.8725	0.1619	−1.018	0.0383	−1.7725	0.0000
*β*	0.0761	0.7165	54.3856	0.3001	7.7506	0.0142
*α*	0.1038	0.7323	5.3444	0.4850	1.4158	0.0357
*γ*	0.0019	0.9711	0.7502	0.0000	0.7900	0.0000
RP						
*μ*	0.0000	0.0459	0.0002	0.0012	−0.0471	0.4581
*φ*	0.0228	0.9555	0.1040	1.1779	0.2118	0.2182
*θ*	−0.0231	0.9551	−0.1045	1.1772	−0.2269	0.1501
*λ*	−5.6948	0.8151	−0.0036	0.8610	1.2088	0.3167
*ω*	−9.2508	0.0412	−6.0945	11.4906	−6.1920	0.1945
*β*	−0.0381	0.6276	2.8587	12.1593	−0.0025	0.4743
*α*	−0.0870	0.6266	−0.6703	2.8912	0.0302	0.6075
*γ*	0.2551	0.0766	−0.3521	0.0579	0.0496	0.7294
TSP						
*μ*	−0.0492	0.5103	0.0000	0.9399	0.0000	0.9615
*φ*	0.5792	0.0041	0.1617	0.3055	0.4073	0.0018
*θ*	−0.4437	0.0849	0.1608	0.3358	−0.1629	0.2672
*λ*	0.0434	0.8788	−0.0039	0.8766	0.0088	0.6918
*ω*	−0.4106	0.9822	−0.0669	0.0770	−0.7030	0.0000
*β*	3.1484	0.8826	0.5660	0.0020	1.3692	0.0696
*α*	0.5642	0.9104	0.0276	0.5786	0.0814	0.5674
*γ*	−0.3110	0.4728	1.0390	0.0000	0.9298	0.0000
Urea						
*μ*	−0.0289	0.1558	0.0103	0.5857	−0.0203	0.1546
*φ*	0.2314	0.3104	0.1280	0.5614	0.1613	0.2675
*θ*	0.1229	0.5871	0.3337	0.1066	0.2554	0.0637
*λ*	0.4986	0.1288	0.0361	0.9026	0.3950	0.0768
*ω*	−0.7959	0.0822	−0.4697	0.0773	−0.7401	0.0053
*β*	0.3482	0.0119	0.1750	0.1000	0.3628	0.0008
*α*	0.0973	0.2649	0.1944	0.0390	0.0998	0.1472
*γ*	0.9026	0.0000	0.9346	0.0000	0.9123	0.0000

The coefficient *λ* in Equation (1) is used to describe the relationship between return and volatility. The statistical significance level is set to 5%. Thus, a *p*-value less than 5% indicates that the estimated coefficient *λ* is statistically different from zero. The full sample spans from December 1985 to October 2017. The first subsample ranges from December 1985 to October 2003, and the second subsample is from November 2003 to December 2017. DAP, PC, RP, and TSP denote diammonium phosphate, potassium chloride, rock phosphate, and triple super phosphate respectively.
